# Is arterial hypotension the real enemy in septic shock or is it just cosmetic?

**DOI:** 10.1016/j.aicoj.2026.100096

**Published:** 2026-06-01

**Authors:** Patrick M. Wieruszewski, Mathieu Jozwiak

**Affiliations:** a Department of Anesthesiology, Mayo Clinic, Rochester, MN, United States of America; b Department of Pharmacy, Mayo Clinic, Rochester, MN, United States of America; c Service de Médecine Intensive Réanimation, CHU de Nice, Nice, France; d UR2CA, Unité de Recherche Clinique Côte d’Azur, Université Côte d’Azur, Nice, France

Circulatory failure is the hallmark pathological consequence of septic shock. Critically low arterial blood pressure during septic shock compromises tissue perfusion and risks irreversible organ injury, complete cardiovascular collapse, and death [1]. Accordingly, prompt resuscitation with fluids and vasopressors has long been recognized as a critical determinant of positive outcome and is unequivocally recommended to commence immediately upon suspicion of sepsis or septic shock [2]. However, while the Surviving Sepsis Campaign recommends targeting a mean arterial pressure of at least 65 mmHg for initial resuscitation, formal recommendations on the rapidity of attaining this target are lacking.

In this issue of *Annals of Intensive Care*, Yokoyama et al. [3] report a post-hoc analysis of Optimal Target Blood Pressure in Elderly with Septic Shock (OPTPRESS), a multicenter, open-label, pragmatic trial of older adults (≥65 years) with septic shock that found greater mortality in those randomized to a higher mean arterial pressure target (80−85 mmHg) compared to a lower target (65−70 mmHg) [4]. In this secondary analysis, Yokoyama et al. sought to evaluate the relationship between duration of arterial hypotension on septic shock presentation and 90-day mortality amongst subjects enrolled in the OPTPRESS trial [3]. They calculated the ‘time-to-target mean arterial pressure’ as the number of hours that lapsed from randomization to first attainment of the OPTPRESS trial group assignment mean arterial pressure target (65 or 80 mmHg). The authors categorized the time-to-target mean arterial pressure based on pragmatic thresholds of 1, 3, 6, and 12 h. In a propensity score adjusted analysis, they found that patients in whom it took at least 12 h to achieve the mean arterial pressure target (or failure to achieve it at all) had greater 90-day mortality than those who achieved it within one hour (OR 1.48, 95% CI 1.11–1.98). Compared to within one hour, none of the other time categories were associated with significantly different odds of mortality. When treated as a continuous variable, there was no association between time-to-target mean arterial pressure and 90-day mortality. In time-to-event analysis when dichotomized at 12 h, delayed time-to-target mean arterial pressure (beyond this threshold or never attained) was associated with over 3-fold greater risk of mortality (HR 3.24, 95% CI 2.19–4.80). Lastly, when compared to within one hour, those who took at least 12 h (or not reached at all) was the only category to have greater risk of mortality (HR 2.93, 95% CI 1.77–4.85). However, all patients who never reached the target (n = 18) died by 90-days, and when they were removed from the at least 12 h group, the effect on death was no longer significant. Their findings suggest that there is a lack of dose-response relationship, but longer time-to-target mean arterial pressure may be an important marker of shock severity. Although, the external validity is limited by the trial conduct restricted to within Japan and in adults at least 65 years of age. Yokoyama et al. should be congratulated on their efforts and thoughtful repurposing of the OPTPRESS trial data. However, certain methodological considerations warrant careful interpretation of the findings, and some clinicians and researchers may still be left wondering, what is the optimal speed at which target mean arterial pressure should be achieved in patients presenting with septic shock?

Arterial blood pressure is a principal determinant of tissue and organ perfusion and has long been recognized as an important resuscitation target in septic shock [2]. However, the way suboptimal mean arterial pressure influences organ injury during septic shock is less clear, making prognostication of arterial hypotension challenging. Intuitively, empirical studies suggest that greater exposure to arterial hypotension is associated with increased odds of acute organ injury and death. Specifically, low mean arterial pressure has been shown to have the strongest relationship with acute organ injury and death, followed by the systolic, diastolic, and pulse pressure [5]. However, the findings of Yokoyama et al. should not be viewed as contradictory to the existing evidence. Their analysis was constrained by the recording of mean arterial pressure in the OPTPRESS trial, which was done so only at prespecified timepoints every four hours from randomization, and not continuously. Thus, it is plausible that target thresholds may have been exceeded in between assessments but not captured in the data recording procedure.

The time-to-target mean arterial pressure only considers the first value exceeding the target threshold and not whether that target was subsequently maintained nor for how long. Indeed, as both the proportion and cumulative time spent below target mean arterial pressure in septic shock increase, the odds of acute kidney injury, myocardial injury, and hospital mortality increase [6]. Yokoyama et al. attempted to assess a similarly quantified hypotension burden, however because of the intermittent mean arterial pressure recordings, their analysis assumes no changes during the 4 h in between measurements, which was unlikely to be true in most subjects. Furthermore, Yokoyama et al. estimated mean arterial pressure using the conventional formula that sums the diastolic pressure and one-third the pulse pressure. While this method is generally reliable in stable patients, calculations such as the harmonic mean, which places greater weight on lower values has been shown to be a more robust approximation of the peripheral mean arterial pressure [7]. Lastly, although the mean arterial pressure is a pragmatic target for bedside titration, it may not truly infer resuscitation status and appropriately indicate adequacy of oxygen delivery, utilization, and thus perfusion at the tissue-level [8].

As we continue to understand the implications of hemodynamic derangement to inform the management strategies for septic shock, the OPTPRESS trial post-hoc analysis by Yokoyama et al. reminds us of the nuances of study design and their potential for influencing interpretation of the findings. While arterial pressure has long been the staple macrocirculatory target, the way in which we quantify its exposure is critical. The findings of Yokoyama et al. suggest that time-to-target mean arterial pressure is an important indicator of shock severity.

## Author contributions

PMW and MJ conceived, drafted, reviewed, and critically revised the manuscript for important intellectual content, and approved the final version.

## Funding

None.

## Declaration of competing interest

PMW receives consulting fees from Wolters Kluwer UpToDate and research funding from ZOLL Foundation. MJ received travel grants from ALAM Medical, is a member of the board of VIATRIS, received fees for lectures for VIATRIS and AOP ORPHAN and is a consultant for AGUETTANT. MJ is a member of the Editorial Board of Annals of Intensive Care.
